# EZH2-Myc Hallmark in Oncovirus/Cytomegalovirus Infections and Cytomegalovirus’ Resemblance to Oncoviruses

**DOI:** 10.3390/cells13060541

**Published:** 2024-03-19

**Authors:** Ranim El Baba, Georges Herbein

**Affiliations:** 1 Department Pathogens & Inflammation-EPILAB EA4266, University of Franche-Comté UFC, 25000 Besançon, France; ranim.elbaba@live.com; 2 Department of Virology, CHU Besançon, 25030 Besançon, France

**Keywords:** oncoviruses, human cytomegalovirus, HCMV, EZH2, Myc, oncogenesis, tumor microenvironment, cytomegalovirus

## Abstract

Approximately 15–20% of global cancer cases are attributed to virus infections. Oncoviruses employ various molecular strategies to enhance replication and persistence. Human cytomegalovirus (HCMV), acting as an initiator or promoter, enables immune evasion, supporting tumor growth. HCMV activates pro-oncogenic pathways within infected cells and direct cellular transformation. Thus, HCMV demonstrates characteristics reminiscent of oncoviruses. Cumulative evidence emphasizes the crucial roles of EZH2 and Myc in oncogenesis and stemness. EZH2 and Myc, pivotal regulators of cellular processes, gain significance in the context of oncoviruses and HCMV infections. This axis becomes a central focus for comprehending the mechanisms driving virus-induced oncogenesis. Elevated EZH2 expression is evident in various cancers, making it a prospective target for cancer therapy. On the other hand, Myc, deregulated in over 50% of human cancers, serves as a potent transcription factor governing cellular processes and contributing to tumorigenesis; Myc activates EZH2 expression and induces global gene expression. The Myc/EZH2 axis plays a critical role in promoting tumor growth in oncoviruses. Considering that HCMV has been shown to manipulate the Myc/EZH2 axis, there is emerging evidence suggesting that HCMV could be regarded as a potential oncovirus due to its ability to exploit this critical pathway implicated in tumorigenesis.

## 1. Introduction

The identification of a particular group of human viruses as the predominant factor behind a significant portion of human cancers stands as a crucial milestone in understanding and addressing cancer. Recent estimates indicate that virus infections account for 15–20% of the global cancer burden [1]. The human oncoviruses that are widely acknowledged include human papillomavirus (HPV), hepatitis B virus (HBV), hepatitis C virus (HCV), Epstein–Barr virus (EBV), Kaposi’s sarcoma-associated herpesvirus (KSHV), human T-cell lymphotropic virus (HTLV-1), and Merkel cell polyomavirus (MCPyV) [2]. In recent findings, the oncogenic potential of human cytomegalovirus (HCMV) has been emphasized, suggesting that the virus has the capability to directly induce transformation in primary cells. This characterization positions HCMV as a potential eighth human oncovirus [3,4]. Certain factors, for instance, the host’s immune response, genetic predisposition, and environmental influences, contribute to the overall cancer risk. Additionally, the concept of viral-induced cancer extends beyond direct viral involvement in cellular transformation. Chronic inflammation induced by certain viruses can create a microenvironment favorable for cancer development [1,5].

The mechanisms by which oncoviruses contribute to oncogenesis are diverse. They may directly influence host cell DNA, disrupt cell cycle regulation, inhibit tumor suppressor genes, or activate oncogenes [6]. Some viruses can also evade the host immune system, allowing for persistent infections that contribute to the accumulation of genetic mutations over time [7]. Human oncogenic viruses exhibit a range of genomes, cellular tropisms, cancer pathologies, and disease prevalences. Despite this diversity, they share common features that can contribute to human cancer development. These viruses can establish chronic infections lasting for years without apparent symptoms [6]. To understand their uniqueness, it is crucial to explore the specific mechanisms through which they modify the cellular environment. Oncoviruses employ various molecular mechanisms to enhance replication and persistence, often involving the hijacking of the host cell signaling machinery, ultimately resulting in the acquisition of cancer hallmarks [8,9,10,11]. During prolonged infection, oncogenic viruses manipulate cellular processes for replication while evading immune detection. As components of their strategies for replication and immune evasion, oncoviruses have developed potent programs with anti-apoptotic and proliferative capabilities, directly triggering cancer hallmarks in the infected cell [12]. On the other hand, HCMV plays a potential role in carcinogenesis by acting as either an initiator or promoter [3]. It enables tumors to evade immune responses by encoding viral proteins and promoting immunosuppressive cellular factors, establishing HCMV-induced immune tolerance that supports tumor growth [3,13]. Recently, it has been shown by our research group that the high-risk oncogenic HCMV strains have the capacity to induce Myc/EZH2 expression and induce the formation of polyploid giant cancer cells (PGCCs), suggesting a potential association between HCMV infection, elevated Myc/EZH2 expression, and polyploidy in vitro and in several tumors [14,15,16,17]. Collectively, our discoveries affirm the suggested tumorigenic properties of Enhancer of zeste homolog 2 (EZH2) and Myc upon HCMV infection.

In this review, we highlighted the significance of EZH2 and Myc oncogenes in oncovirus and HCMV infections, and focused on indicating that HCMV might be classified as a potential oncovirus. This classification stems from HCMV’s capability to utilize the essential EZH2/Myc axis associated with tumorigenesis.

## 2. EZH2 and Myc: Key Players in Cancers

Cumulative evidence underscores the pivotal roles of EZH2 and Myc in both oncogenesis and stemness [18]. EZH2, a member of the polycomb group genes (PcGs), is a crucial epigenetic regulator within the polycomb repressive complex 2 (PRC2). Additionally, EZH2 can methylate non-histone targets independently of PRC2 or interact with certain proteins in order to activate downstream genes [19]. EZH2 serves as a master regulator in the progression of cell cycling, autophagy, and apoptosis, while also promoting DNA damage repair, inhibiting cellular senescence, and influencing the determination of cell lineage as well as signaling pathways. The diverse roles of EZH2 in various biological processes contribute to its association with numerous diseases, notably cancer [18,20]. Studies provide evidence of elevated EZH2 expression in various cancer types, such as breast cancer, glioblastoma, ovarian cancer, gastric cancer, prostate cancer, esophageal cancer, anaplastic thyroid carcinoma, and nasopharyngeal carcinoma [14,15,16,17,19]. Due to its significant involvement in cancer pathophysiology, EZH2 emerges as a prospective target for treating cancer [19,20]. Various EZH2 inhibitors have been formulated, and multiple clinical trials are ongoing to assess the effectiveness of drugs targeting EZH2 across diverse cancer types [20].

The Myc family oncogene undergoes deregulation in over 50% of human cancers often linked to poor prognosis (for instance, glioblastoma, high-grade lymphoma, and acute leukemia). Research has uncovered a role for Myc in therapy resistance in multiple cancer types including neuroblastoma and small-cell lung carcinoma [21,22]. Myc oncoproteins, functioning as potent transcription factors, play a critical role in governing various cellular processes, contributing to tumorigenesis [21]. Activated Myc mechanisms entail either the intrinsic acquisition of cancer hallmarks within the cells or the disruption of the tumor microenvironment and host immune responses. Elevated expression of Myc and/or mutated Myc have been observed in the majority of human cancers [23]. Myc particularly controls gene expression and regulates cellular proliferation and differentiation, and cell cycling, metabolism, as well as apoptosis [22]. Further, it induces the expression of EZH2 through activating the EZH2 promoter, directly suppressing miR-137, or inhibiting miR-26a [15,24]. In addition, the protein Bromodomain-4 (BRD4) regulates EZH2 transcription positively through Myc upregulation [25].

In glioblastoma stem cells (GSCs), co-immunoprecipitation experiments revealed that STAT3 co-precipitated not only with EZH2 but also with SUZ12 and EED, which are core components of PRC2. This suggests a potential interaction between STAT3 and PRC2 [26]. EZH2 in brain tumors is influenced by various molecular pathways, including long non-coding RNAs (lncRNAs) (such as HOTAIR, AGAP2-AS1, NEAT1, LINC-PINT, PAR5, PVT1, LINC00963, HOXB13-AS1, PART1, and SNGH7) as well as microRNAs (miRNAs) (such as miR-144, miR-454-3p, miR-124a, miR-133b, miR-524-5p, miR-324-5p, and miR-138) [27]. To exert its enzymatic activity, EZH2 forms a complex with non-catalytic proteins like EED, SUZ12, and RbAp48/46 [27]. Its COOH-terminus contains a SET domain responsible for its methyltransferase activity [28]. With a unique structure consisting of four distinct domains, the SET and CXC domains mediate histone methyltransferase activity, the two SANT domains enable DNA binding for chromatin remodeling and transcriptional modulation, and ncRBD facilitates interaction with non-coding RNAs (ncRNAs) [28,29,30]. Beyond its canonical function of histone H3 methylation, EZH2 methylates non-histone substrates like STAT3 and Jarid2, influencing their transcriptional activities. EZH2 can methylate retinoic acid receptor-related orphan receptor alpha (RORα) and promyelocytic leukemia zinc finger protein (PLZF), leading to the degradation of these target proteins [31]. In estrogen receptor-negative breast cancer, EZH2 generates a complex with RelA and RelB, activating NF-kB signaling without involving methylation [32]. Recent human cancer genome sequencing has uncovered frequent mutations in genes that encode the subunits of SWI/SNF chromatin remodeling complexes across various cancers. EZH2 expression was observed in the majority of cancer cell lines and xenografts possessing mutations of the SWI/SNF subunits AT-rich interaction domain 1A (ARID1A); polybromo-1 (PBRM1); and SWI/SNF-related, matrix-associated, actin-dependent regulator of chromatin, subfamily A, member 4 (SMARCA4), which are some of the most commonly mutated SWI/SNF subunits in human cancer [33]. In castration-resistant prostate cancer, EZH2 interacts with the androgen receptor, utilizing a methylation-dependent mechanism for gene expression activation [34]. In breast cancer, the oncoprotein-binding domain of Yin Yang 1 (YY1) has the ability to attract EZH2, leading to the downregulation of both phosphatase and tensin homolog (PTEN) and PTENP1. The collaboration between YY1 and EZH2 hinders the transcription of PTEN and PTENP1, subsequently enhancing Akt phosphorylation at S473 and T308 and increasing the activation of Akt [35].

Myc’s core function lies in transcription, facilitated by various protein–protein interactions (PPIs) with the general transcriptional machinery. Such interactions are crucial for Myc’s role in regulating transcription [36]. Myc, along with other transcription-associated machinery (the positive transcription elongation factor b, P-TEFb; BRD4; mediator 1, MED1; and super elongation complex, SEC, proteins), exploits these interactions to drive transcription [36,37]. The initial PPIs discovered involve preserved regions within Myc, such as the transactivation domain (TAD), the basic region (BR), the helix–loop–helix–leucine zipper (HLH–LZ) domain, and Myc homology boxes (MBs), which are preserved among species and the Myc family oncoproteins as well. For instance, Myc interacts with Myc-associated factor X (MAX) via the respective HLH–LZ regions, enabling DNA binding through the BRs on both proteins, and is crucial for Myc’s overall function [38,39]. Other structurally identified Myc interactors include WD repeat-containing protein 5 (WDR5) with the MBIIIb region, TATA box-binding protein (TBP) with an amino terminus, bridging integrator 1 (BIN1) via MBI, and Aurora kinase A (AURKA) via MBI and certain flanking regions on Myc-N [36,40]. Moreover, the binding of peptidyl-prolyl cis–trans isomerase PIN1 to the amino-terminal MB0 illustrates the various low-affinity interactions within the PIN1–MYC complex [41]. These interactors collectively contribute to Myc’s role as a transcription factor and a potent oncoprotein [36].

In cases of acute leukemia, EZH2 exhibits noncanonical roles through binding to Myc at sites outside the PRC2 targets. It utilizes a concealed TAD for recruiting co-activators and activating genes. Both the canonical (EZH2-PRC2) and noncanonical (EZH2-TAD-Myc-coactivators) functions of EZH2 contribute to oncogenesis, elucidating the limited and gradual efficacy of inhibitors targeting EZH2’s catalytic function in anti-tumor effects (Table 1) [37,42]. Through competitive binding with the SKP1–cullin-1–F-box complex that contains FBW7 as the F-box protein (SCF^FBW7^) ubiquitin ligase, EZH2 opposes the FBW7-mediated polyubiquitination of Myc, preventing its proteasomal degradation. Depleting EZH2, rather than inhibiting its enzymatic activity, results in substantial degradation of Myc, leading to the inhibition of tumor growth in Myc-driven small-cell lung carcinoma and neuroblastoma [18]. In bladder cancer, BRD4 positively influences EZH2 transcription by upregulating Myc [25]. In summary, these studies highlighted the link between EZH2, Myc, and tumor progression.

## 3. Activation of EZH2-Myc Axis by Oncoviruses

This section, on the activation of the “EZH2-Myc axis by oncoviruses”, delves into the intricate molecular interactions between EZH2 and Myc, key regulators in cellular processes, within the context of oncovirus infections (Table 2). This axis has emerged as a crucial focal point in understanding the mechanisms underlying viral-induced oncogenesis. This exploration aims to unravel the specific roles of EZH2 and Myc in well-established oncoviruses, shedding light on their contributions to the initiation and development of virus-associated malignancies.

To start with, high-risk human papillomavirus (HR-HPV) can influence histone methylation through various machineries. Dysregulated H3K27me is a prevalent histone modification in various tumors, including HPV-positive cancers [43]. High-grade cervical intraepithelial lesions positive for HPV16 exhibited elevated EZH2 levels. HPV E6 and E7 promoted EZH2 expression; HPV E6 increased the levels of the transcription factor Forkhead box protein M1 (FOXM1); while HPV E7 activated E2 promoter binding factor 1 (E2F1) by binding to retinoblastoma (Rb). It is worth noting that FOXM1 is a transcription factor required for a wide spectrum of essential biological functions, including DNA damage repair, cell proliferation, cell cycle progression, cell renewal, cell differentiation, and tissue homeostasis. Additionally, it was observed that p53 represses EZH2 expression, suggesting that an E6-mediated loss of p53 may lead to increased EZH2 expression [44]. Certainly, HTLV-Tax, rather than HTLV-1 bZIP factor (HBZ), enhanced EZH2 promoter activity through a mitogen-activated protein kinase (MAPK)- and nuclear factor kappa B (NFκB)-dependent mechanism, leading to elevated EZH2 protein levels. In addition, the study demonstrated that inhibiting EZH2 hindered Tax-induced growth and the immortalization of Tax-transfected peripheral blood mononuclear cells (PBMCs) [45]. The induction of EZH2 by KSHV was crucial for the initiation of KSHV-induced angiogenesis. Elevated levels of EZH2 were detected in Kaposi sarcoma tumors. In vitro, the latent KSHV infection increased EZH2 expression in human endothelial cells by activating the NF-κB pathway through viral FLICE-inhibitory protein (vFLIP) and latency-associated nuclear antigen (LANA), which are considered two KSHV-latent genes. The upregulation of EZH2 by KSHV was essential for inducing Ephrin-B2, a key proangiogenic factor that stimulates the formation of endothelial cell tubules. Thus, findings suggested that KSHV manipulates the host epigenetic regulator EZH2 to enhance angiogenesis [46,47].

In the context of HBV, HBx elevates EZH2 expression by diminishing miR-101 levels, a microRNA targeting EZH2 transcripts, and inhibiting Rb, leading to E2F1-mediated transcription of the EZH2 gene [43,48]. The overexpression of Rb resulted in cell arrest within the G1 phase, while Rb-deficient cells exhibited an accelerated G1 transition, supporting the notion of Rb as an inhibitor of cell proliferation [49]. Beyond its involvement in cell cycle regulation, Rb was linked to the control of numerous cellular functions, such as DNA replication, differentiation, as well as apoptosis. The reduced potential for differentiation and heightened rates of proliferation observed in Rb-deficient cells could both play a role in tumorigenesis [49]. It is worth noting that following aberrant mitosis, Aurora B phosphorylates Rb at serine 780, which negatively regulates endoreduplication by enhancing Rb’s interaction with E2F1. This interaction prevents the activation of the E2F1 promoter and the consequent formation of polyploid cells [50]. Moreover, EZH2 was identified as a target of miR-124, and a notable negative correlation between EZH2 mRNA levels and miR-124 was observed in hepatocellular carcinoma (HCC) tissues. This discovery indicates that the HCV core protein may influence H3K27me3 levels via the miR-124/EZH2 pathway [51].

The expression of EZH2 was elevated in primary B cells upon EBV infection, highlighting its significance in viral infections. Similar to intercellular adhesion molecule 1 (ICAM1), it is speculated that the EZH2 gene might also be induced by NF-κB activation from latent membrane protein 1 (LMP1), as NF-κB activation has been documented to stimulate EZH2 gene expression [52]. A study showed that knocking out the EZH2 gene led to heightened EBV gene expression within the lytic phase, consequently facilitating viral replication and progeny production. The EBV genes analyzed, such as latent membrane proteins (LMPs), EBV nuclear antigens (EBNAs), EBV-encoded RNAs (EBER1), Bam-HI A rightward transcripts (BARTs), and lytic genes, exhibited significantly higher expression in the knockout (KO) cells compared to the wild-type cells following lytic induction. Remarkably, lytic and latent genes showed a robust response to EZH2 knockout after lytic induction. Such findings indicated that EZH2 plays a role in suppressing the expression of latent viral genes throughout the lytic phase [52]. Both Merkel cell polyomavirus (MCV) large T (LT) antigen and HPV E7 oncoproteins contain a specific amino acid motif (leucine(L)-x-cysteine(C)-x-glutamine(E)) that binds to and inhibits Rb, leading to malignant cell transformation. The inhibition of Rb by viral oncoproteins triggers the activation of E2F transcription factors, promoting cellular progression from the G1 phase to the S phase of the cell cycle [53]. Several studies have shown that E2F downstream targets, for instance, survivin and SRY (sex determining region Y)-box 2, also known as SOX2, which is a transcription factor that is essential for maintaining the self-renewal, or pluripotency, of undifferentiated embryonic stem cells, are activated in HPV- and MCV-associated tumors [54,55,56]. It has been identified that EZH2, which is known to be regulated by E2F, is usually activated by MCV large T (LT) antigen and HPV E7 in MCV-positive and HPV16-positive cancer cells [53]. Another study revealed that EZH2 and EED play a crucial role in cell proliferation, serving as vital downstream effectors of E2F activity. Collectively, these results indicated that EZH2 and embryonic ectoderm development (EED) are natural targets of the RB-E2F pathway, and that the dysregulation of this pathway, a common occurrence in human tumors, would lead to elevated EZH2 and EED levels [57]. Notably, in Merkel cell carcinoma (MCC) tissues, EZH2 exhibited high expression. Studies showed that the expression of viral MCV LT antigen is necessary for EZH2 mRNA expression, and that EZH2 shows a crucial role in the growth of MCV-positive MCC cells [53]. Thus, targeting the direct expression of the EZH2 protein emerges as a promising approach for inhibiting cancer growth in MCV-positive MCC patients.

**Figure 1:**
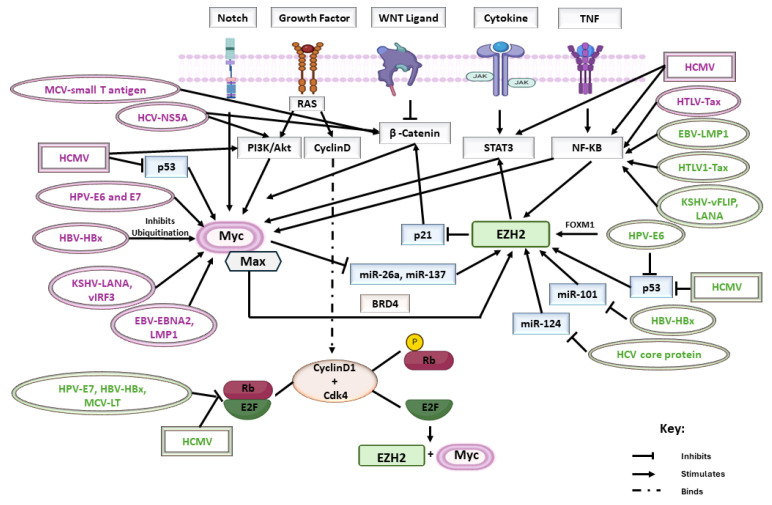
Impact of oncoviruses and HCMV on Myc and EZH2 expression. The influence of oncoviruses and HCMV on Myc and EZH2 expression involves a complex interplay within the host cellular environment. Oncoviruses and HCMV can modulate the levels of Myc and EZH2, two key molecular players, through various mechanisms. These mechanisms may include direct interaction with cellular machinery, activation of signaling pathways, or manipulation of host transcriptional and epigenetic regulation. The virus names highlighted in purple target Myc pathways whereas the ones highlighted in green target EZH2 pathways. Oval shapes represent all oncoviruses while the rectangular shapes signify HCMV.

The expression of the Myc oncogene plays an essential role in the intricate interplay between oncoviruses and host cells, influencing various cellular processes and contributing to the pathogenesis of virus-associated cancers [23] (Figure 1). HPV E6 as well as E7 target Myc, which is identified as a marker protein in various cancers, including cervical cancer. Upon disruption by E6/E7, Myc interrupts cell proliferation, apoptosis, as well as cellular transformation probably due to the HPV genome’s integration within the Myc locus [58]. The dysregulation of c-myc results in the disturbance of Cdks, cyclins, as well as E2F transcription factors. Myc has the ability to induce cyclin/Cdk complexes through Cdk activating kinase (CAK) and Cdc25 phosphatases. Myc is identified to counteract the Cdk-inhibitory function of p21 and p27 [59]. Both E6 and E7 bind to c-Myc, leading to the activation of the human telomerase reverse transcriptase (hTERT) promoter and thereby leading to cancer cell immortality. hTERT is a catalytic enzyme that is required for telomerase activity (TA) and cancer progression. hTERT promoter activation by E6 necessitates Myc binding sites (E boxes) on the hTERT promoter. Thus, Myc and E6 are found on the activated promoter [60,61]. Even in latency, KSHV sustains elevated Myc expression, a critical factor for maintaining a latent status and promoting cell survival. Various viral latent proteins, including LANA and viral IFN regulatory factor-3 (vIRF3), have been demonstrated to stabilize Myc at the post-translational phase [62].

It was well established that both EBNA2 and LMP1 have the capacity to induce Myc expression. EBNA2 plays a pivotal role in initiating the immortalization process and this is by promptly and directly activating cellular target genes, including Myc. The regulation of Myc by EBNA2 involves the induction of chromatin loops that connect two enhancers upstream of the Myc transcriptional starting site. By directly activating Myc expression, EBNA2 is implicated in various events that trigger B-cell activation, proliferation, and survival [63]. Tax is recognized for inducing the transcription of Myc and specificity protein 1 (SP1), and this is by activating NF-κB [64]. In an in vivo setting, using a transgenic murine model that expressed the complete HCV open reading frame, elevated Myc expression was validated, indicating a direct involvement of HCV protein expression in c-Myc induction. The activation of Akt by the HCV non-structural protein NS5A, along with the stabilization of β-catenin, was identified as the mechanism responsible for activating the c-Myc promoter and increasing c-Myc transcription [65]. A previous investigation demonstrated that the hepatitis B virus X protein (HBx) enhances the stability of Myc through a mechanism that inhibits Myc ubiquitination [66]. The small T antigen of polyomavirus activates genes by stimulating Myc synthesis and stabilization; it disrupts WNT signaling by elevating the primary key regulator of the pathway, beta-catenin, which plays a role in the induction of Myc [67].

Myc oncoprotein exhibits characteristics that extend beyond its direct role in transformation, contributing to genomic instability by inducing reactive oxygen species (ROS), and causing DNA breaks and chromosome instability, resulting in tetraploidy and aneuploidy [68]. Modifications to cell cycle checkpoints play a vital role in specific forms of genomic instability, including gene amplification. c-Myc stimulated entry into the S-phase and contributed to the formation of endomitosis, polyploidy, and neoplastic cellular transformation [69]. Notably, HTLV-1 p30II uniquely amplifies the transforming activity of c-Myc, promoting S-phase progression and polyploidy. This enhancement is achieved by the interaction with Myc-associated transcriptional coactivators transformation/transcription domain-associated protein (TRRAP)/p434 and the histone acetyltransferase TIP60, resulting in the stabilization of HTLV-1 p30II/Myc-TIP60 chromatin-remodeling complexes [69]. An analysis of transcriptomic networks revealed compromised PRC2 activity in triple-negative breast cancer (TNBC) despite increased EZH2 levels. The inverse association between PRC2 activity and EZH2 is accompanied by elevated hypoxia-inducible-factor 1A (HIF1A) and FOXM1. Interestingly, HIF1-α inhibition on PRC2 did not impact EZH2, unveiling dual antagonistic functions of HIF1-α in influencing EZH2 activity in response to hypoxia and contributing to aggressive TNBC development [70].

## 4. Activation of EZH2-Myc Axis by HCMV

As previously mentioned, accumulating evidence underscores the pivotal roles of Myc and EZH2 in both oncogenesis and stemness. Following HCMV infection of human mammary epithelial cells (HMECs), ovarian epithelial cells (OECs), and astrocytes, a subtle activation of Myc and EZH2 as well as polyploidy induction were observed [15,16,71]. Myc overexpression can induce DNA replication, potentially leading to polyploidy [72]. An intricate relationship between cancer, Myc, and polyploidy has been established, with Myc being associated with nuclear pleomorphism in renal cell carcinomas [73]. This axis’ activation was notably detected in CMV-transformed HMECs (CTH), CMV-transformed OECs (CTO cells), and CMV-elicited glioblastoma cells (CEGBCs) exhibiting a significant increase in Myc and EZH2 proteins. The aforementioned cells were generated upon HCMV infection of HMECs, OECs, and HAs with high-risk oncogenic HCMV-DB and BL strains and strains isolated from breast cancer, high-grade serous ovarian carcinoma, and glioblastoma biopsies [14,15,16,71,74].

Several studies confirmed that HCMV infection has the potential to trigger Myc induction, foster stemness, and initiate pro-EZH2 pathways [14,15,16]. Interestingly, previous findings revealed that high-risk HCMV-BL and DB induced Myc expression, coupled with low p53 levels, which could stimulate the replicative potential of stem cells as well as progenitor reprogramming in breast cancer [14,71]. Myc was recognized as a transcriptional target of p53 in mammary stem cells and was stimulated upon p53 loss. Furthermore, HCMV-DB significantly increased pRb (phosphorylated retinoblastoma) expression, with the RB-E2F pathway described to regulate EZH2 expression [14]. The EZH2/Myc axis was considered one of the effective mechanisms in characterizing and classifying the HCMV strains into high-risk (HR) and low-risk (LR) HCMV strains. To start with, HCMV-DB and HCMV-BL were categorized as HR strains due to their ability to transform HMECs, OECs, and HAs in vitro [14,15,16,75]. Similarly, HCMV strains isolated from TNBC, glioblastoma (GBM), and high-grade serous ovarian carcinoma (HGSOC) biopsies demonstrated the transformation of HMECs, OECs, and HAs, respectively. At the molecular level, these HR strains were classified by elevated expression of Myc and EZH2, activation of the phosphotylinosital 3 kinase (PI3K)/Protein kinase B (Akt) pathway, and repression of the p53 and Rb genes. Conversely, LR-HCMV strains did not promote a sustained expression of Myc, EZH2, or Akt following the acute infection of HMECs, OECs, and Has, leading to cellular death in chronically infected cultures. Further, LR-HCMV strains did not induce the elevation of cellular markers that were involved in oncogenesis, as observed with HR-HCMV strains, especially in tumors associated with poor prognosis [14,15,16,71]. Interestingly, isolates of high-risk HCMV strains obtained from patients with TNBC, GBM, and HGSOC exhibited similar transformative capabilities. In these cell cultures, the presence of PGCCs was observed, which demonstrated high Myc and EZH2 levels, suggesting a potential association between HCMV, EZH2, and Myc and polyploidy. CTO and CTH cells underwent dedifferentiation, possessing traits of stemness and epithelial–mesenchymal transition (EMT) [14,15,16,71,75]. Conversely, CEGBCs underwent dedifferentiation and exhibited characteristics of stemness, proneural mesenchymal transition (PMT), as well as features indicative of invasiveness [15].

## 5. Impact of EZH2 and Myc on Immunity

EZH2 and Myc are both important regulators of gene expression and play significant roles in immune cell fate. In the context of tumors, EZH2 is often overexpressed and associated with aggressive cancer phenotypes, metastasis, and poor prognosis, where it promotes tumor growth by silencing tumor suppressor genes and dysregulating various signaling pathways [76]. In immune cells, EZH2 has been shown to regulate differentiation. It is crucial for the development and function of regulatory T cells (Tregs), which suppress immune responses and promote immune tolerance. Moreover, EZH2 can influence the differentiation and function of other immune cell types, such as CD8+ T cells and dendritic cells, although its effects may vary depending on the context [77]. In cases of chronic antigen stimulation, for instance, in cancer and viral infections, the stem cell-like progenitor population characterized by TCF-1 expression exhibits traits including self-renewal, the capacity to generate effector cells, and a heightened responsiveness to PD-1 blockade. Studies in acute viral models have demonstrated that the absence of EZH2 leads to an elevated expression of memory-related transcripts like TCF-7, which is associated with the stem-like CD8+ T cells observed in cancer. It is intriguing to consider that precise timing of EZH2 inhibition could potentially enlarge reservoirs of stem-like CD8+ T cells, thereby fostering larger pools of effector cells, and consequently boosting both anti-tumor and anti-viral immunity [78]. Myc can influence the behavior of tumor-infiltrating immune cells, such as T cells and macrophages, by modulating the expression of genes involved in immune regulation, inflammation, and immune evasion. It can promote the differentiation and activation of effector T cells, enhancing anti-tumor immune responses. However, it can also contribute to T-cell exhaustion and dysfunction in the tumor microenvironment [79]. T-cell exhaustion, a chief condition, is observed in human immunodeficiency virus (HIV), HBV, HCV infections, and cancer [80]. Myc has been shown to regulate the metabolism of immune cells, influencing their function and fate. For example, Myc-driven metabolic reprogramming can promote the differentiation of effector T cells and support their function in the tumor microenvironment [81].

**Figure 2:**
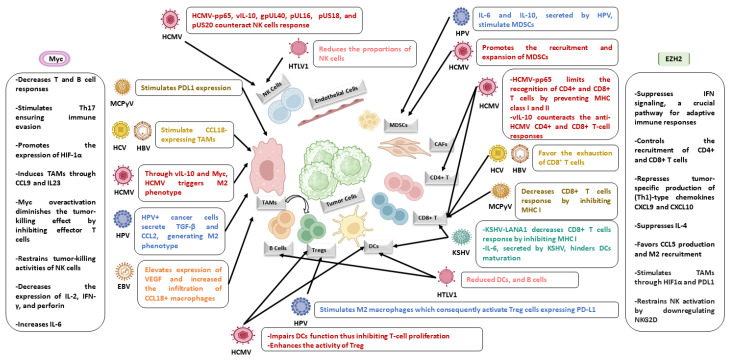
EZH2 and Myc expression along with oncoviruses and HCMV play a crucial role in shaping an immunosuppressive tumor microenvironment. The EZH2/Myc-mediated interplay between the host and cancer in the presence of oncoviruses or HCMV establishes a microenvironment favorable to cancer cells—facilitating growth, invasion, migration, angiogenesis, and aiding in evading anti-tumor immune responses. Abbreviations: TAMs, tumor-associated macrophages; NK, natural killer cells; MDSCs, myeloid-derived suppressor cells; CAFs, cancer-associated fibroblasts; DCs, dendritic cells; Tregs, regulatory T cells; Th17, T helper 17; HIF-α, hypoxia inducible factor-alpha; IL, interleukin; IFN-γ, interferon-gamma; PDL1, programmed cell death ligand 1; vIL10, viral interleukin 10; TGF-β, transforming growth factor-beta; VEGF, vascular endothelial growth factor; MHC, major histocompatibility complex; NKG2D, natural-killer group 2, member D.

EZH2 plays two key roles in regulating the immune response within tumor cells. Firstly, it contributes to maintaining chronic inflammation, and secondly, it establishes immune tolerance within the tumor microenvironment (TME). Chronic inflammation can promote epigenetic changes and induce oncogenesis. On the contrary, genetic and epigenetic alterations in tumor cells can induce an inflammatory microenvironment that supports cancer progression. EZH2 is involved in transcriptional activation of pro-inflammatory genes, for instance, interlukin-6 (IL-6)/tumor necrosis factor (TNF), and repression of interferon-γ receptor 1 (IFNGR1), promoting tumorigenesis through chronic inflammation [82,83]. The EZH2-STAT3 signaling axis is implicated in chronic inflammation-related cancers. In the TME, chronic immune cells both promote oncogenic activities and fail to mount an effective anti-tumor immune response. In metastatic prostate cancers, EZH2 represses IFNGR1 expression in a Myc-dependent manner, impairing interferon (IFN) signaling, which is crucial for anti-tumor immunity [84]. In ovarian and colorectal cancers, EZH2 negatively correlates with tumor-infiltrating CD8+ T cells as well as patient outcomes. EZH2-mediated epigenetic changes repress chemokines CXCL9 and CXCL10, hindering effector T-cell trafficking to the TME. Epigenetic modulator therapy increases T-cell infiltration, decelerates tumor progression, and enhances the therapeutic efficacy of programmed death-ligand 1 (PD-L1) checkpoint blockade and adoptive T-cell transfusion. In melanoma, anti-CTLA-4 or IL-2 immunotherapy causes T-cell infiltration and TNF-α production in the TME, resulting in EZH2 upregulation. This upregulation silences immune-related genes, causing treatment resistance. Thus, combining EZH2 inhibition with immunotherapy improves T-cell infiltration, promoting IFN-γ-producing CD8+ T cells in the TME and enhancing tumor control [82,85]. In addition, EZH2 has been reported to suppress IFN signaling, a crucial pathway for antigen presentation and adaptive immune responses. EZH2 limits natural killer (NK) cell activation by reducing the expression of natural-killer group 2, member D (NKG2D) ligands (Figure 2). EZH2 directly binds to the promoters of NK cell ligands, UL16 binding protein 1 (ULBP1) and MHC class I polypeptide–related sequence A (MICA). Thus, blocking EZH2 leads to an improved elimination of HCC by NK cells [82,86].

Moreover, the elevated Myc expression in the TME significantly influences cancer development through diverse mechanisms. Within tumor cells, studies revealed that Myc-dependent pathways indirectly impact the expression of cytokines in the TME. This includes a decrease in the expression of IL-2, IFN-γ, and perforin, coupled with an increase in IL-6. For instance, nuclear AURKA enhances PD-L1 expression via a Myc-dependent pathway, inhibiting IFN-γ expression and facilitating immune evasion by tumors [87]. Myc interacts with other signaling pathways, notably the WNT/β-catenin pathway, providing robust support for tumor progression in various cancers like colorectal, breast, and hepatocellular carcinoma [88,89,90]. On the other hand, within immune cells and the TME, Myc plays a role in immune-cell-related events that inhibit innate and adaptive immune responses (Figure 2). Myc overactivation in innate immune cell subsets hampers proinflammatory mediator production, elevates immunosuppressive cytokine production, and restrains the tumor-killing activities of effector cells, for instance NK cells [87]. In adaptive immune cells, Myc overactivation diminishes the tumor-killing effect by inhibiting effector T cells. It controls CD47 and PD-L1 expression in diverse tumor cells, impacting immune checkpoint pathways; the overexpression of these molecules driven by Myc inhibits both innate and adaptive immune responses, thereby promoting tumor progression [91]. All in all, Myc’s effects on immune activities promote tumor proliferation by influencing both innate and adaptive immunity. The transcription factor Myc, which plays roles in cell proliferation, apoptosis, tissue remodeling, angiogenesis, cell metabolism, and cytokine production, appears to share common pathways with macrophages [92]. Indeed, research by Pello et al. indicates that Myc expression is observed in human-derived macrophages categorized as M2 macrophages, known for their anti-inflammatory functions, involvement in angiogenesis, and tissue remodeling [93].

## 6. Oncoviruses and HCMV Foster a Pro-Oncogenic Environment in the Presence of EZH2 and Myc

In numerous human tumors, such as those affecting the breast, renal, prostate, bladder, lung, cervical carcinoma, lymphoma, glioma, and melanoma, an elevated presence of tumor-associated macrophages (TAMs) within the tumor stroma is correlated with an increased likelihood of metastasis and the development of more aggressive cancer types [94]. TAMs play a role in various processes such as angiogenesis (secretion of vascular endothelial growth factor, VEGF; transforming growth factor beta, TGF-β; matrix metalloproteinases, MMPs), migration, and invasion (secretion of MMPs; epidermal growth factor, EGF; serine proteases), EMT (secretion of TGF-β), intravasation and extravasation (production of CCL18 chemokine), and interaction with cancer stem cells, and ultimately contribute to immunosuppression (expression of PD-L1/PD-L2, production of IL-10, TGF-β, arginase-1, and prostaglandins) [95,96]. It is noteworthy to highlight that within human macrophages, the expression of Myc is restricted to the M2 phenotype. Conversely, Myc is not observed in resting (M0) or pro-inflammatory (M1) macrophages. Myc promoted the expression of VEGF, MMP9, HIF-1α, and TGF-β in TAMs [93]. Reports indicated that TAMs in neuroblastomas are linked to the upregulation of Myc expression through the IL-6/STAT3 pathway [97]. This suggests the existence of potential feedback loop mechanisms involved in the regulation of Myc expression within the TME. Ongoing studies highlighted the impact of EZH2 in tumor cells on TAMs via H3K27 methylation. For instance, the miR-144/miR-451a cluster, silenced by EZH2, promotes M1 polarization of TAMs, enhancing anti-tumor immunity in hepatocellular carcinoma. Functionally, hepatic miR-144/miR-451a stimulates the anti-tumor immune response of infiltrating macrophages through paracrine signaling. These miRNAs form a feedback loop with EZH2, which is implicated in promoting HCC progression by epigenetically suppressing tumor suppressor genes. In contrast to prior findings suggesting that miR-144/miR-451a inhibit HCC cell proliferation by targeting oncogenes such as EZH2, the abovementioned study revealed their predominant tumor-suppressive role in reshaping the tumor immune microenvironment. These differences may arise from HCC heterogeneity or variations in experimental methodologies, including the use of cell lines with diverse gene expression profiles or intracellular signaling contexts [98]. EZH2 influences macrophage infiltration in small-cell lung cancer by mediating H3K27me3 in the enhancer region of CCL2. In lung cancer, EZH2 favors CCL5 production, recruiting M2 macrophages, thus enabling metastasis and macrophage infiltration [99,100]. In tumor cells, miRNAs interacting with EZH2 contribute to macrophage polarization. For example, in gliomas, EZH2-mediated inhibition of miRNA-454-3p promotes M2 macrophage polarization [101]. Additionally, EZH2 in invasive breast cancer regulates LOXL4 through miR-29b/miR-30d, influencing macrophage infiltration and extracellular matrix remodeling. EZH2 inhibition attenuates M2-related gene expression (Arg1 and CD206) and enhances M1-related gene expression (TNF-α; nitric oxide synthase 2, Nos2; and IL-6) [102,103].

Recent findings indicated that Myc plays a significant role in regulating the EBV lytic switch. EBV employs a strategy of switching between latent and lytic programs to evade immune surveillance. Myc has been observed to bind to the EBV genome’s origin of lytic replication and inhibit its interaction with the lytic cycle initiator BZLF1 promoter. Interestingly, the abundance of Myc diminishes during plasma cell differentiation, which is a critical trigger for lytic reactivation [104]. EBV seropositivity was found to be associated with higher counts of total TAMs and CCL18-expressing TAMs. The injection of CNE2 (NPC cell line)-EBV-positive cells into humanized mice resulted in elevated expression of VEGF and the cytokine granulocyte macrophage-colony stimulating factor (GM-CSF), increased infiltration of CCL18+ macrophages, the emergence of an EMT-like phenotype, and elevated rates of lung metastasis when administered subcutaneously [105,106]. Within the HPV+ TME, M2 macrophages are predominant. The HPV+ cancer cells secrete TGF-β and CCL2, leading to the differentiation of macrophages into the M2 phenotype. Consequently, M2 macrophages inhibit M1 macrophages and activate Treg cells. Treg cells express PD-L1, which binds to its receptor PD-1, inducing apoptosis in lymphocyte T (CD8 cells). Moreover, M2 macrophages secrete TGF-β, IL-10, and IL-6, creating a feedback loop on their stimulation. Furthermore, TGF-β and EGF induce EMT. IL-6 and IL-10 stimulate myeloid-derived suppressor cells (MDSCs), akin to M2 macrophages, which then secrete arginase-1 (Arg-1) and inducible nitric oxide synthase (iNOS) [107,108]. TAMs, by secreting IL-10, promote T lymphocytes’ differentiation into regulatory T lymphocytes. Further, IL-6 was found in the tumor microenvironment of Kaposi’s sarcoma (KS) and the peripheral circulation of individuals with KSHV-associated tumors [109]. In addition to its involvement in promoting cell migration and angiogenesis, IL-6 may hinder dendritic cell maturation, potentially suppressing immune activation specific to the virus or tumor. Moreover, IL-6 may encourage B-cell expansion, potentially increasing the pool of B-cell targets for the expansion of the viral reservoir within the microenvironment of KSHV-associated tumors [110,111]. KSHV LANA1 inhibits major histocompatibility complex class I (MHC I) peptide presentation as a strategy for immune evasion [112]. A distinct M2 subpopulation characterized by elevated expression of CCL18 was predominantly detected in advanced HCC. Findings indicated that early-stage HCC exhibits a higher percentage of cytotoxic CD8+ T cells, demonstrating robust cytotoxicity. In contrast, advanced HCC displays an increased proportion of exhausted CD8+ T cells and a reduced percentage of cytotoxic CD8+ T cells with diminished killing capacity. Due to the increased infiltration of M2 macrophages and Treg cells in advanced HCC, the anti-tumor efficacy of cytotoxic CD8+ T cells is further compromised [113]. In the context of HTLV1, TAM infiltration is frequently observed in acute and lymphoma-type adult T-cell leukemia (ATL), correlating with an unfavorable prognosis. In patients with ATL, there is a reduction in the proportions of NK cells and dendritic cells in the peripheral blood. Single-cell analyses have revealed a concurrent reduction in B cells and an elevation in myeloid cells in ATL. These myeloid cells also exhibit elevated expression of activation markers like CD64 and immune checkpoint molecules, for instance, PD-1 [114]. Moreover, MCC cell lines show a reduction in HLA class I expression [115]. In seven of eight MCPyV-positive samples, PD-L1 expression was found by tumor cells. The tumor cells were enclosed by PD-L1/CD33 immune cells. The expression of PD-L1 by tumor cells was higher in areas having denser immune infiltrates [116].

Similar to oncoviruses, HCMV creates an immunosuppressive environment with cytokines (viral IL-10 and TGF-β) and oncogenes (EZH2 and Myc) potentially facilitating oncogenic transformation [3,4,13,14,15,16,117,118]. In the presence of viral interlukin-10 (vIL-10) and Myc, HCMV promotes macrophage reprogramming into an M2 phenotype in the tumor microenvironment, resembling TAMs. The highly macrophage-tropic HCMV-DB strain shifts infected macrophages towards an M2/TAM phenotype, linking specific clinical strains with oncogenesis [118]. HCMV-DB infection of macrophages favors an M2/TAM phenotype parallel to the upregulation of the proto-oncogene Bcl3 [119]. The STAT3 pathway and VEGF were activated by HCMV, promoting angiogenesis. In addition, vIL-10 and US28 boost cancer cell invasion and metastasis [118]. The prolonged survival of neutrophils and mononuclear cells in the TME supports oncogenesis by activating an angiogenic switch [13]. Collectively, HCMV fosters a pro-oncogenic environment, facilitating cancer progression by activating cancer stem cells, angiogenesis, invasion, and an EMT phenotype [13,118]. Moreover, lncRNAs have been identified as effective contributors, where they facilitate signal transductions within tumor signaling pathways and promote tumor evasion from immune surveillance. Studies have demonstrated that various immune cells, including T and B cells, macrophages, dendritic cells, and myeloid cells, regulate tumor immune responses through pathways associated with lncRNAs [120,121]. Within CTH cells, HCMV lncRNA4.9 was previously detected in tumors obtained from xenograft NSG mice injected with CTH cells, as well as in biopsies from breast cancer [122]. Additionally, the suppression/mutation of p53 has been demonstrated to result in decreased MHC-I presentation, STAT3 phosphorylation, increased PD-L1 expression facilitated by microRNA (miR34), enhanced production of pro-inflammatory chemokines and cytokines, and an indirect upregulation of CXCR4 and CXCR5. Rb loss led to an increase in CCL2 and IL6 secretion, attributed to elevated fatty acid oxidation (FAO) activity and enhanced mitochondrial superoxide (MS) production [13]. These molecular alterations, induced by HCMV, have been associated with immune suppression within the TME (Figure 2).

## 7. Comparative Oncogenic Traits: HCMV-Resembling Oncoviruses

HCMV exhibits characteristics similar to those of oncoviruses, which could have implications for understanding its potential contributions to oncogenesis. It raises questions about whether HCMV shares molecular mechanisms or strategies with oncoviruses in promoting cancer development. HCMV activates EZH2 and Myc in a manner analogous to the processes seen in oncovirus infections. This implies a shared molecular mechanism in the manipulation of these crucial cellular components. To distinguish between high- and low-risk HCMV strains, it is crucial to characterize the involved viral strains, the biological features of the infected cell, and its microenvironment both in vitro and in vivo [4]. HCMV-DB and HCMV-BL have been classified as high-risk HCMV strains as they showed a sustained transformation of acutely infected HMECs, OECs, and HAs in vitro, resulting in the formation of CTH cells, CTO cells, and CEGBCs, respectively [14,15,16,71,74,75,123]. PGCCs as well as cellular heterogeneity were detected only upon infection with high-risk HCMV strains. In contrast, the low-risk (HCMV-KM and FS) strains revealed no transforming potentials and caused cell death in long-term cultures [15,16,71,123].

At the molecular level, an EZH2^High^ Myc^High^ molecular profile was detected with high-risk strains in CTH cells, CTO cells, and CEGBCs compared to low-risk strains [15,16,71,123]. Interestingly, EZH2 and Myc were mainly expressed in the PGCC subpopulation of CTO-DB and BL cultures [16]. In line with these data, we previously detected clinical HCMV strains in breast cancer, HGSOC, and GBM biopsies with elevated EZH2 and Myc expression [14,15,16]. To start with, two HCMV strains isolated from TNBC biopsies, namely HCMV-B544 and -B693, transformed HMECs toward CTH cells and PGCCs in vitro; these strains triggered the enhanced expression of cellular markers actively involved in oncogenesis such as Myc, Akt, and EZH2 [14,75]. From HGSOC biopsies, three clinical HCMV strains were isolated. These strains induced the transformation of OECs, resulting in the generation of CTO cells with elevated proliferative capabilities, accompanied by increased expression of EZH2/Myc and the formation of PGCCs. Notably, inhibiting EZH2 led to a reduction in these features, indicating the influence of EZH2 on the observed cellular transformations [16]. In the context of GBM, HCMV-GBM strains isolated from GBM biopsies induced a CEGBC phenotype exhibiting upregulated EZH2 and Myc with tumor heterogeneity, proneural-to-mesenchymal plasticity, as well as stemness resulting in spheroid formation and invasiveness [15]. High-risk HCMV strains, like other oncoviruses, induced EZH2 and Myc expression, potentially contributing to the development and progression of cancer. The shared molecular and cellular phenotypes highlight and strengthen the similarities between high-risk HCMV and recognized oncoviruses, prompting a re-evaluation of its classification.

## 8. Conclusive Thoughts

In conclusion, the importance of EZH2 and Myc oncogenes in characterizing viral strains lies in their central role in the molecular and cellular events leading to virus-induced oncogenesis. The available findings revealed the crucial association between oncoviruses, EZH2, and Myc in the context of several malignancies’ progression. Hence, this axis underscores the importance of exploring epigenetic modifications and oncogene interactions in the context of viral infections, offering potential avenues for targeted therapeutic interventions and novel diagnostic strategies. Finally, HCMV exhibited numerous characteristics of oncoviruses within both infected cells and the surrounding tissue microenvironment. This suggests a strong association between HCMV and the oncovirus family that extends beyond its known oncomodulatory effects.

## Figures and Tables

**Table 1:** Inhibitors targeting EZH2 and Myc.

EZH2/PRC2 Inhibitors		Myc Inhibitors	
Inhibitor Name	Target	Inhibitor Name	Target
Tazemetostat, EL1, GSK126, CPI-169, EPZ005687, EPZ011989, ZLD10A, GSK503, JQEZ5, GSK926, GSK343, PF-06726304, MS1943, EZH2-IN-3, CPI-1205, EBI-2511, UNC1999, valemetostat, (R)-OR-S1, PF-06821497, oxetinib (AZD9291)	**EZH2**	QN-1, APTO-253, AZD5153, GSK525762, dBET1	**Myc transcription**
		MLN0128, silvestrol, eFT226, BTYNB	**Myc translation**
		Pyrimidine, derivatives, SZL-P1–41, TD19, volasertib	**Myc stability**
		MYCMI-6, KI-MS2-008, Omomyc, FPPa-OmoMYC	**Myc-Max heterodimer**
PROTAC EED Degrader-1, PROTAC EED Degrader-2, UNC6852	**EED**	Sulfopin, ASH2L-derived peptides, C620-0696	**Accessibility of Myc to downstream genes**

**Table 2:** Impact of oncoviruses as well as HCMV on EZH2 and Myc.

Viral Pathogen	EZH2		Myc	
	Involved Oncogene(s)	EZH2 Interactions	Involved Oncogene(s)	Myc Interactions
HPV	HPV E6	-Increased the levels of the transcription factor FOXM1 and promoted EZH2 and H3K27me3 expression -E6-mediated loss of p53 led to increased EZH2 expression	HPV E6 and HPV E7	-E6 as well as E7 interacted with c-Myc, leading to the activation of the hTERT promoter -Activation of the hTERT promoter by E6 necessitates Myc binding sites (E boxes) on the hTERT promoter -E7 formed a complex with the myc-interacting zinc-finger protein-1 (Miz-1)
	HPV E7	-Activated E2F1 by binding to Rb, thus promoting EZH2 and H3K27me3 expression		
HTLV	Tax	-MAPK- and NFκB-dependent mechanism led to elevated EZH2 protein levels	Tax	-Induced the transcription of Myc by activating NF-κB
KSHV	vFLIP and LANA	-Activated the NF-κB pathway leading to increased expression of EZH2	LANA and vIRF3	-Stabilized Myc at the post-translational level
HBV	HBx	-Elevated EZH2 expression by diminishing miR-101 -Inhibited Rb, resulting in E2F1-mediated transcription of the EZH2 gene	HBx	-Inhibited the ubiquitination of Myc
HCV	HCV core protein	-Influenced H3K27me3 levels through a miR-124/EZH2 pathway	HCV non-structural protein NS5A	-Activated Akt -Stabilization of the transcription factor β-catenin, thus activating the c-Myc promoter and increasing c-Myc transcription
EBV	LMP1	-NF-κB activation stimulated EZH2 gene expression	EBNA2 and LMP1	-EBNA2 induced chromatin loops that connect two enhancers upstream of the Myc transcriptional starting site -LMP1 activated NF-κB; NF-κB is a positive regulator of Myc expression
MCPyV	MCV LT	-Inhibition of Rb triggered the activation of E2F transcription factor and EZH2 expression	small T antigen	-Disrupted WNT signaling by elevating the primary key regulator of the β-catenin pathway, thus stimulating Myc synthesis and stabilization
HCMV	IE1, IE2, pUL97	-Increased phosphorylated-Rb (pRb) through the pRB-E2F pathway described to regulate EZH2 expression	IE1, IE2	-Low p53 levels -Increased Myc, Fos, and Jun expression by IE

## Abbreviations

ATL	Adult T-cell leukemia
Arg-1	Arginase-1
ARID1A	AT-rich interaction domain 1A
AURKA	Aurora kinase A
BARTs	Bam-HI A rightward transcripts
BR	Basic region
BIN1	Bridging integrator 1
BRD4	Bromodomain-4
CAK	Cdk-activating kinase
CEGBCs	CMV-elicited glioblastoma cells
CTH	CMV-transformed HMECs
CTO cells	CMV-transformed OECs
E2F1	E2 promoter binding factor 1
EBER1	EBV-encoded RNAs
EBNAs	EBV nuclear antigens
EED	Embryonic ectoderm development
(EZH2	Enhancer of zeste homolog 2
EMT	Epithelial–mesenchymal transition
EBV	Epstein–Barr virus
FAO	Fatty acid oxidation
FOXM1	Forkhead box protein M1
GBM	Glioblastoma
GSCs	Glioblastoma stem cells
GM-CSF	Granulocyte macrophage-colony stimulating factor
HLH–LZ	Helix–loop–helix–leucine zipper
HBV	Hepatitis B virus
HBx	Hepatitis B virus X protein
HCV	Hepatitis C virus
HGSOC	High-grade serous ovarian carcinoma
HR	High-risk
HR-HPV	High-risk human papillomavirus
HBZ	HTLV-1 bZIP factor
HCMV	Human cytomegalovirus
HIV	Human immunodeficiency virus
HMECs	Human mammary epithelial cells
HPV	Human papillomavirus
HTLV-1	Human T-cell lymphotropic virus
hTERT	Human telomerase reverse transcriptase
HIF1A	Hypoxia-inducible-factor 1A
iNOS	Inducible nitric oxide synthase
ICAM1	Intercellular adhesion molecule 1
IFN	Interferon
IFNGR1	Interferon-γ receptor 1
IL-6	Interlukin-6
KSHV	Kaposi’s sarcoma-associated herpesvirus
KS	Kaposi’s sarcoma
KO	Knockout
LT	Large T
LANA	Latency-associated nuclear antigen
LMP1	Latent membrane protein 1
LMPs	Latent membrane proteins
lncRNAs	Long non-coding RNAs
LR	Low-risk
MHC I	Major histocompatibility complex class I
MMPs	Matrix metalloproteinases
MED1	Mediator 1
MCC	Merkel cell carcinoma
MCPyV	Merkel cell polyomavirus
MCV	Merkel cell polyomavirus
LT	Large T
MICA	MHC class I polypeptide–related sequence A
miRNAs	MicroRNAs
MS	Mitochondrial superoxide
MAPK	Mitogen-activated protein kinase
E boxes	Myc binding sites
MBs	Myc homology boxes
MAX	Myc-associated factor X
Miz-1	Myc-interacting zinc-finger protein-1
MDSCs	Myeloid-derived suppressor cells
NK	Natural killer
NKG2D	Natural-killer group 2, member D
Nos2	Nitric oxide synthase 2
ncRNAs	Non-coding RNAs
NFκB	Nuclear factor kappa B
OECs	Ovarian epithelial cells
PBMCs	Peripheral blood mononuclear cells
PTEN	Phosphatase and tensin homolog
pRb	Phosphorylated retinoblastoma
PI3K	Phosphotylinosital 3 kinase
Akt	Protein kinase B
PBRM1	Polybromo-1
PcGs	Polycomb group genes
PRC2	Polycomb repressive complex 2
PGCCs	Polyploid giant cancer cells
P-TEFb	Positive transcription elongation factor b
PD-L1	Programmed death-ligand 1
PLZF	Promyelocytic leukemia zinc finger protein
(PMT	Proneural mesenchymal transition
PPIs	Protein–protein interactions
ROS	Reactive oxygen species
Tregs	Regulatory T cells
Rb	Retinoblastoma
RORα	Retinoic acid receptor-related orphan receptor alpha
SOX2	Sex-determining region Y-box 2, also known as
SCFFBW7	SKP1–cullin-1–F-box complex that contains FBW7 as the F-box protein
SP1	Specificity protein 1
SEC	Super elongation complex
SMARCA4	SWI/SNF-related, matrix-associated, actin-dependent regulator of chromatin, subfamily A, member 4
Th17	T helper 17
TBP	TATA box-binding protein
TA	Telomerase activity
TAD	Transactivation domain
TRRAP	Transformation/transcription domain-associated protein
TGF-β	Transforming growth factor beta
TNBC	Triple-negative breast cancer
TME	Tumor microenvironment
TNF	Tumor necrosis factor
TAMs	Tumor-associated macrophages
ULBP1	UL16 binding protein 1
VEGF	Vascular endothelial growth factor
vFLIP	Viral FLICE-inhibitory protein
vIRF3	Viral IFN regulatory factor-3
vIL-10	Viral interlukin-10
WDR5	WD repeat-containing protein 5
YY1	Yin Yang 1
